# Focal Task-Specific Dystonia: An M1-Centered TSMS Framework

**DOI:** 10.3390/brainsci16101011

**Published:** 2026-09-24

**Authors:** Norman Lu, Rodrigo F. O. Pena

**Affiliations:** Department of Biological Sciences, Florida Atlantic University, Jupiter, FL 33458, USA; normankwlu@gmail.com

**Keywords:** focal dystonia, musician’s dystonia, rehabilitation, neuroplasticity, motor retraining, computational modeling

## Abstract

**Highlights:**

**What are the main findings?**

- An M1 task-specific motor synergy with recurrent excitation, disproportionately strong relative to rapid PV-centered inhibition, is proposed as the initiating abnormality in FTSD.
- Altered movement coordination is hypothesized to produce a reduced-capacity state of task-specific output limitation, termed true weakness; subsequent overreaching may then drive excitatory-biased plasticity and formation of a symptom-threshold-dependent dystonic synergy.

**What are the implications of the main findings?**

- Under specified model assumptions, an input-dependent firing-rate crossover is interpreted as supporting an E/I-balance mechanism, while other parameter combinations can produce the same behavior and structural contributions require further investigation.
- Below- or at-threshold retraining (BATR) is proposed as a testable strategy to raise the symptom-threshold through accurate practice within the intact functional range.

**Abstract:**

Focal task-specific dystonia (FTSD) is an isolated dystonia in which abnormal muscle contractions occur only during a particular motor task while most other movements remain relatively spared. In this hypothesis article, we propose that FTSD arises within a task-specific motor synergy (TSMS) in primary motor cortex (M1), a local excitatory–inhibitory ensemble supporting a specific movement component of the affected task. We hypothesize that excitatory synapses within the affected TSMS become disproportionately strong relative to parvalbumin (PV)-centered inhibitory circuitry, creating a hyperexcitable dystonic synergy recruited when attempted task intensity exceeds the capacity of the residual functional synergy. A change in movement coordination is proposed to reduce the capacity of a previously established TSMS, producing a state of task-specific output limitation, termed true weakness, at higher intensities. Repeated overreaching beyond this reduced capacity may then favor excitatory over inhibitory strengthening, thereby driving the emergence and progressive consolidation of the dystonic synergy and generating the symptom-threshold phenomenon. Under the imposed architecture and parameterization, a spiking neural network exhibited an input-dependent crossover in the relative firing rates of specified functional and E/I-imbalanced model populations. We interpret these results as supporting an E/I-balance mechanism for the crossover, while recognizing that other parameter combinations can produce the same behavior, consistent with degeneracy. Structural contributions require further investigation to distinguish these possibilities. Finally, we propose below- or at-threshold retraining (BATR), in which symptom-free practice may promote inhibitory rebalancing and raise the symptom-threshold. This framework offers an experimentally testable M1-centered account of FTSD.

## 1. Introduction

### 1.1. Background and Rationale

Focal task-specific dystonia (FTSD) is an isolated dystonia in which abnormal muscle contractions are elicited during a particular motor activity or task while most other movements remain relatively spared [1,2,3]. Because symptoms may remain restricted to one task or task condition, FTSD provides a useful model for investigating maladaptive sensorimotor plasticity [4]. Neurophysiological studies have repeatedly reported reduced short-interval intracortical inhibition (SICI) and evidence consistent with primary motor cortex (M1) hyperexcitability in FTSD [5,6,7]. These findings support a disturbance of rapid intracortical inhibitory control, but they do not by themselves identify the initiating anatomical site, the responsible interneuron class, or the causal sequence through which FTSD develops.

Basal ganglia-, cerebellar-, and network-centered accounts each organize important aspects of the dystonia literature, including altered pathway signaling, abnormal motor calibration, and distributed motor-system dysfunction [8,9]. Nevertheless, the conjunction of marked task specificity, relative preservation of other movements, and intensity-dependent symptom expression remains only partially explained at the level of a specific motor-generating circuit.

In this hypothesis article, we advance an M1-centered account in which the relevant unit of dysfunction is a task-specific motor synergy (TSMS): a local excitatory–inhibitory ensemble in M1 that supports a particular unidirectional movement component within an affected task. We hypothesize that excitatory synaptic strength within the affected TSMS becomes disproportionately strong relative to rapid inhibitory control centered on parvalbumin-expressing (PV) interneurons. This proposed imbalance would create a hyperexcitable dystonic synergy that becomes preferentially recruited when task demands exceed the capacity of the residual functional synergy. Reduced SICI and disturbed surround inhibition are consistent with impaired rapid cortical inhibition, but current human TMS methods do not provide cell-type- or synapse-specific resolution. PV involvement is therefore treated here as a mechanistic inference and experimentally testable hypothesis rather than as a directly established cellular lesion.

We further propose a candidate etiological pathway in which a substantial change in the spatiotemporal coordination of a previously learned movement reduces the effective excitatory and inhibitory capacities of the corresponding TSMS. Repeated attempts to perform above this reduced capacity are hypothesized to generate fragmented or poorly coordinated cortical activity that strengthens excitatory connections without equivalent strengthening of inhibitory circuitry. Over time, this asymmetric plasticity may give rise to and progressively consolidate a dystonic synergy, producing the characteristic symptom-threshold phenomenon. Predisposing biological factors may influence susceptibility to this process. For example, altered cortical inhibition in non-manifesting DYT1 carriers indicates that abnormal excitability can exist without overt dystonia, consistent with the possibility that physiological predisposition may be insufficient by itself for clinical expression [10].

FTSD is also associated with abnormalities outside M1, including altered basal ganglia signaling, cerebellar connectivity and modulation, somatosensory processing, and spinal inhibition [8,11,12,13]. The existence of these abnormalities is not disputed, but their temporal and causal relationship to cortical dysfunction remains unresolved. The present framework hypothesizes that at least some extra-M1 findings arise or become reinforced through repeated activation of an abnormal M1 synergy. A companion hypothesis article, *Focal Task-Specific Dystonia Beyond M1: Network Adaptations and a Causal-Architecture Taxonomy of Dystonia*, develops these proposed extra-M1 network adaptations in greater detail. The proposed causal ordering should be understood as a testable prediction rather than as an established conclusion.

Current treatments for FTSD include botulinum toxin injections, oral medications, task modification, rehabilitative interventions, and, in selected severe cases, surgery [3,14]. Although these approaches can provide symptomatic benefit, they are not designed to test the proposed TSMS-level mechanism. Reports of slow-down exercise and related sensorimotor retraining suggest that carefully constrained practice can improve performance, although the evidence remains limited and the responsible mechanisms are unresolved [15,16,17]. Building on this clinical precedent, we formulate BATR as a mechanism-informed rehabilitation hypothesis in which symptom-free practice is predicted to promote E/I rebalancing within the affected TSMS.

### 1.2. Threshold Phenomenon and Key Terminology

Many FTSD phenotypes exhibit a symptom-threshold phenomenon in which the affected task can be performed without overt dystonic symptoms at lower movement intensities or under specific symptom-sparing task conditions, whereas dystonic contractions emerge once task demands exceed a particular level [15,17,18,19]. We use symptom-threshold to denote the highest task intensity, under a specified performance condition, at which overt dystonic symptoms remain absent. The symptom-threshold is a behavioral construct whose defining movement parameters are task-specific and may consist of a single dominant variable, such as speed or force, or a multidimensional combination of movement demands.

This term is distinct from spike threshold, or firing threshold, which denotes the membrane potential level at which an individual neuron generates an action potential. We therefore hyphenate symptom-threshold when referring to the behavioral phenomenon and use spike threshold when referring to neuronal firing. The computational crossover introduced later is defined separately and is treated as a proposed qualitative analogue of the behavioral symptom-threshold rather than as a directly calibrated equivalent. The principal terms used throughout the proposed TSMS framework are summarized in Table 1.

### 1.3. Scope and Objectives

This hypothesis article synthesizes published evidence; it presents no original human-participant or animal data and does not report a clinical case. The article has four principal aims. First, it proposes an M1-centered mechanism in which FTSD arises from an imbalance between excitatory pyramidal circuitry and PV-centered inhibitory control within an affected TSMS. Second, it outlines a candidate etiological sequence linking altered movement coordination and repeated overreaching to the formation of an excitatory-dominant dystonic synergy. Third, it formulates BATR as a testable rehabilitation hypothesis. Fourth, it uses a spiking neural network model to test whether an imposed excitation–inhibition (E/I) difference can generate an input-dependent crossover between specified functional and E/I-imbalanced model conditions. The model addresses only this restricted network-level proposition and does not establish M1 localization, PV specificity, the proposed etiological sequence, or BATR efficacy. The framework is intended to generate falsifiable predictions that can be compared with basal ganglia-primary, cerebellar-primary, and distributed-network alternatives.

## 2. The Proposed M1-Centered TSMS Framework

### 2.1. Core Pathophysiological Hypothesis: PV-Centered E/I Imbalance Within an M1 TSMS

#### 2.1.1. Definition and Proposed Organization of a TSMS

Motor synergies are commonly described at a macro level as coordinated patterns of muscle activation that support a movement or motor subtask [20]. We retain the term synergy because a particular unidirectional movement component at an effector may be generated through the coordinated recruitment of several muscles or muscle groups. Here, however, we use the term task-specific motor synergy (TSMS) in a narrower, hypothesis-level sense to denote a task-dependent ensemble of pyramidal neurons and local inhibitory interneurons within the relevant M1 representation that contributes to this coordinated output. For example, a finger-flexion TSMS may contribute to the coordinated recruitment of both intrinsic hand and extrinsic forearm muscles that routinely act together to flex the relevant digit. Proximal stabilizers and muscles that influence posture without directly generating the specified movement direction are treated as interacting with, but not constituting the primary output of, the TSMS.

A muscle may participate in more than one TSMS when it contributes to several movement directions, and individual cortical neurons may participate in multiple, partially overlapping TSMSs. The TSMS is therefore proposed as a task-dependent functional microcircuit rather than a discrete anatomical module or a one-to-one mapping among neurons, muscles, and movements. Its organization is hypothesized to emerge through repeated co-recruitment of excitatory and inhibitory elements during performance of a learned movement component, making it the circuit-level unit in which the proposed FTSD-related E/I imbalance is instantiated.

#### 2.1.2. PV-Centered Inhibitory Control in a Functional TSMS

Across the neocortex, including M1, nearly all GABAergic interneurons fall within three non-overlapping molecular families: parvalbumin-positive (PV), somatostatin-positive (SST), and ionotropic serotonin receptor 5HT3a-expressing (5HT3aR) interneurons [21]. We focus on PV fast-spiking interneurons because their rapid, predominantly GABA_A_-mediated perisomatic inhibition is well positioned to regulate the timing, gain, and spatial specificity of pyramidal population activity [22]. SST and 5HT3aR interneurons may also contribute to TSMS function and are considered separately below.

Within a functional TSMS, increasing pyramidal activity is expected to recruit matched local inhibitory circuitry. Excitatory input onto inhibitory neurons (E→I) drives rapid feedback inhibition, while inhibitory output onto pyramidal neurons (I→E) limits excessive or spatially diffuse firing. This recurrent interaction is conceptually analogous to pyramidal-interneuron network gamma, or PING-like, coupling, although the TSMS hypothesis does not require a particular gamma-oscillatory regime [23].

Motor-skill learning is accompanied by experience-dependent remodeling of excitatory and inhibitory circuitry in M1, including strengthening of horizontal intracortical connections, LTP-like modification and occlusion, subtype-specific reorganization of inhibitory contacts, and phase-dependent changes in glutamatergic and GABAergic transmission [24,25,26,27]. Coordinated plasticity across excitatory and inhibitory inputs can also regulate the local E/I relationship in other cortical preparations [28,29]. On this basis, we propose that E-E, E→I, and I→E motifs within a functional TSMS become jointly adapted during skill acquisition, allowing output to scale while rapid inhibitory control and movement specificity are preserved. Existing evidence supports experience-dependent E/I remodeling, but proportional co-adaptation of E-E, E→I, and I→E motifs within a human TSMS remains a hypothesis.

#### 2.1.3. Neurophysiological Evidence and Cell-Type Inference

In FTSD, we hypothesize that recurrent excitation and rapid local inhibition become mismatched within the affected TSMS. Specifically, E-E connectivity is proposed to become disproportionately strong relative to the E→I and/or I→E motifs that recruit and deliver fast inhibitory control. This constitutes a relative circuit imbalance rather than a complete loss of inhibition: PV-centered circuitry may remain functional but fail to scale adequately as excitatory drive and task demand increase. The imbalance could therefore reflect insufficient recruitment of PV interneurons, reduced efficacy of their inhibitory output, or both.

A substantial TMS literature in FTSD and related focal dystonias reports reduced SICI (e.g., [5,6,7,30,31,32]). Because SICI is strongly influenced by rapid GABAA receptor-mediated intracortical inhibition, reduced SICI is consistent with deficient fast inhibitory control in M1 [33]. However, SICI does not directly quantify E→I or I→E strength within a TSMS or identify PV interneurons. The PV-centered interpretation therefore remains a biologically motivated hypothesis based on the convergence of the SICI phenotype, the rapid time course of the inhibitory abnormality, and the fast-spiking, perisomatic role of PV interneurons.

Findings for long-interval intracortical inhibition (LICI) are more heterogeneous: LICI has been reported as normal in some cohorts [5,34], reduced in others [35,36], and occasionally increased [37]. This contrast with the more frequently reported reduction in SICI suggests that rapid and slower inhibitory processes are not affected uniformly across FTSD phenotypes. It does not exclude SST or 5HT3aR involvement, but it motivates the comparison below between deficient rapid perisomatic inhibition and disturbances of slower dendritic or disinhibitory pathways.

#### 2.1.4. Alternative Interneuron Classes and Phenomenological Predictions

A useful qualitative comparison can be drawn between the predicted consequences of disturbances in rapid perisomatic inhibition and those of disturbances in slower dendritic or disinhibitory pathways. Shao and Burkhalter [38] have demonstrated that slow GABA_B_ receptor-mediated inhibition can constrain late polysynaptic activity: blocking this inhibition in rat visual cortex unveiled reverberant excitatory responses following an initial stimulus. Because dendrite-targeting SST/Martinotti-cell motifs recur across neocortical areas, including M1, these findings motivate the possibility that a predominant reduction of slower dendritic inhibition in a motor circuit would prolong or destabilize recurrent excitation after an initial motor command [39].

In an idealized TSMS, such a disturbance might be expected to favor delayed, reverberant, or temporally fragmented activity rather than solely increasing the immediate gain of the initial pyramidal response. This prediction differs qualitatively from many FTSD presentations, in which the affected task elicits sustained or patterned co-contraction once the symptom-threshold is crossed. The comparison is nevertheless inferential rather than diagnostic. The relevant evidence was obtained outside human M1; GABA_B_-mediated inhibition cannot be assigned uniquely to one interneuron population; and FTSD itself can include mixtures of tonic, phasic, and tremulous features. Clinical phenomenology therefore cannot exclude SST involvement or establish interneuron identity by itself.

By contrast, a predominant reduction of rapid PV-centered perisomatic inhibition, with slower dendritic inhibitory mechanisms remaining relatively preserved, would be expected to increase the probability and spatial extent of the initial pyramidal population response. Continued recurrent excitation could then sustain an elevated output state while slower inhibitory processes limit late reverberation. This configuration provides a plausible qualitative account of a task-bound contraction that emerges when input exceeds a particular intensity and remains present during continued performance. It does not demonstrate that PV dysfunction is sufficient to cause biological FTSD, but it identifies a network phenotype that is consistent with the proposed PV-centered mechanism.

A similar qualification applies to 5HT3aR interneurons, including vasoactive-intestinal-peptide-positive (VIP) populations that frequently inhibit SST interneurons and thereby regulate dendritic disinhibition. Reduced VIP output could enhance SST-mediated inhibition and decrease pyramidal gain, whereas excessive VIP recruitment could reduce dendritic inhibition and facilitate recurrent excitation. These effects are strongly state- and circuit-dependent, however, and cannot be mapped uniquely onto a particular clinical movement phenotype. The present framework therefore does not require SST or VIP circuitry to be entirely normal. Rather, it proposes that deficient rapid PV-centered inhibitory control is the dominant abnormality responsible for the core threshold-dependent motor output, while SST- and 5HT3aR-related pathways may modify its expression.

This comparison generates a testable prediction. The selective weakening of PV-centered inhibition within an experimentally defined motor ensemble should reproduce threshold-dependent excessive and sustained output more consistently than comparable disruption of SST- or VIP-centered pathways. Conversely, the selective restoration of PV recruitment or output should increase the input level at which abnormal activity emerges. Direct cell-type-specific manipulation and recording will be required to evaluate these predictions.

#### 2.1.5. Surround Inhibition and Motor-Output Consequences

Within the proposed framework, dystonic output can be recruited through direct or indirect routes. In the direct case, the affected TSMS itself is driven above its symptom-threshold. At lower attempted intensities, residual rapid inhibition may remain sufficient to constrain pyramidal output; as attempted intensity increases, excitatory recruitment is hypothesized to outpace the available inhibitory control, pushing the TSMS into a hyperexcitable regime that produces involuntary contraction, posturing, or other dystonic output.

In the indirect case, strong activation of a neighboring TSMS generates excitatory activity that spreads toward the affected representation. Surround inhibition would normally suppress this competing output, but a deficient inhibitory boundary may allow the spillover to recruit the affected TSMS into its dystonic regime. Direct symptom expression therefore reflects insufficient control when the affected TSMS itself is strongly recruited, whereas surround-inhibition failure reflects inadequate suppression of that TSMS during recruitment of a neighboring representation.

This indirect route offers a candidate explanation for the disturbed surround inhibition reported in focal hand dystonia [40,41,42]. Rapid local inhibition is well positioned to suppress competing cortical outputs, and deficient PV-centered control could facilitate spillover into an affected TSMS. Human surround inhibition is nevertheless an emergent systems-level phenomenon that may involve several cortical and subcortical processes; its proposed dependence on PV-centered synaptic weakness is therefore a prediction of the framework rather than an established cellular explanation.

More generally, at the synaptic level, the proposed TSMS E/I imbalance could arise through reduced excitatory recruitment of inhibitory neurons (E→I), reduced inhibitory efficacy onto pyramidal neurons (I→E), or both. Reduced SICI is compatible with diminished net GABA_A_-mediated inhibition but does not distinguish these loci. Experimental disruption of local GABA_A_-mediated inhibition nevertheless demonstrates that cortical disinhibition can degrade motor selectivity. In nonhuman primates, focal bicuculline application to motor or premotor cortex produced abnormal motor output, including inappropriate agonist–antagonist co-contraction [43]. In humans with focal dystonia, peripheral afferent input that normally suppresses motor cortical output has instead been reported to facilitate it, supporting abnormal inhibitory gating of sensorimotor input [44]. These observations support the idea of a contribution of insufficient rapid inhibition in M1 without identifying a particular interneuron class or synaptic locus.

I-wave studies provide an additional, indirect probe of this circuitry. In healthy M1, a subthreshold conditioning pulse can suppress later I-wave components more strongly than the earliest I1 component, indicating differential intracortical regulation of the indirect volleys contributing to corticospinal output [45]. Current direction also biases recruitment of partly distinct cortical circuits: posterior-anterior stimulation commonly favors earlier I-wave recruitment, whereas anterior-posterior stimulation can place greater weight on later indirect-wave circuitry [46].

In focal hand dystonia, Hanajima et al. [47] reported reduced SICI under one current orientation while inhibition under another was comparatively preserved. This pattern indicates that intracortical inhibition is altered in a circuit- and orientation-dependent manner rather than uniformly absent. Similarly, the greater conditioning-pulse intensity sometimes required to elicit SICI suggests reduced recruitability or an altered operating state of the inhibitory network [32]. These findings are compatible with the insufficient E→I recruitment of rapid inhibitory circuitry, although altered interneuron excitability, differences in the excitatory populations sampled by TMS, and broader network-state changes remain possible.

Taken together, the evidence supports three relatively secure conclusions: rapid intracortical inhibition is frequently altered in FTSD; surround inhibition and sensorimotor gating are disturbed in at least some phenotypes; and experimentally reducing local GABA_A_-mediated inhibition in the motor cortex can produce abnormal co-contraction and loss of motor selectivity. The more specific claim advanced here is that these findings arise from insufficiently strengthened PV-centered E→I and/or I→E circuitry relative to E-E connectivity within an affected TSMS. That claim remains a mechanistic hypothesis. If correct, cell-type-specific measures should reveal deficient PV recruitment or output within the affected motor ensemble, and restoration of that inhibitory control should expand the range of task intensity over which normal motor output is maintained.

### 2.2. Proposed Etiological Pathway: From Altered Movement Coordination to Dystonic Synergy

#### 2.2.1. Reorganization of a Previously Learned TSMS

The sequence developed below proposes how FTSD could arise following a substantial alteration in the spatiotemporal coordination of a previously learned movement. Such an alteration may result from a deliberate technique change or from another, potentially subtle or unrecognized, change in how an established task is executed. Within this specific etiological pathway, altered coordination is the hypothesized initiating trigger and is therefore necessary to the sequence as formulated, but it is not proposed to be sufficient by itself to produce dystonia. Instead, it is hypothesized to reduce functional TSMS capacity and create the conditions under which repeated overreaching becomes maladaptive. Predisposing biological factors are treated as susceptibility modifiers, whereas repeated overreaching beyond the resulting reduced capacity is hypothesized to drive the excitatory-biased plasticity that gives rise to and progressively consolidates an E/I-imbalanced dystonic synergy. Once formed, repeated above-threshold recruitment and wider network adaptations may maintain or reinforce symptom expression.

When a learned task is executed with a substantially different coordination pattern, the new movement is unlikely to recruit a wholly separate population of M1 neurons. Motor cortical populations are broadly tuned to movement direction, muscle combinations, and other movement features, and individual neurons can contribute to multiple, partially overlapping ensembles ([48] and references therein; [49]). The prior and altered coordination patterns are therefore expected to share some neuronal and synaptic components while differing in others.

At the movement level, this overlap may reflect shared directional, muscular, or joint-related features. Two methods of producing the same piano keystroke, for example, may differ in global hand posture, force distribution, and interdigit coordination while preserving flexion of a particular digit or stabilization of neighboring fingers. Cortical populations contributing to these shared components may continue to be recruited, whereas populations and temporal relationships supporting features unique to the prior pattern may be recruited differently.

At the microcircuit level, the overlap is hypothesized to include E-E connectivity together with associated E→I and I→E motifs. Because pyramidal neurons and inhibitory interneurons may participate in multiple task-dependent ensembles, some previously strengthened connections may remain appropriate for the altered movement and continue to be recruited. The prior and altered TSMSs should therefore be understood as partially overlapping functional configurations rather than anatomically separate networks dedicated to an “old” and a “new” technique. Connections whose recruitment and timing remain compatible with the altered coordination may retain or further strengthen their efficacy, whereas connections optimized for relationships no longer present may become susceptible to weakening.

#### 2.2.2. Plasticity During Altered Movement Coordination

A central premise of the proposed pathway is that learned synaptic efficacy is instantiated at particular connections and activity relationships rather than being freely transferable between motor patterns. Long-term changes in excitatory efficacy can involve postsynaptic receptor trafficking and presynaptic modification, while motor training in M1 is accompanied by the formation and selective stabilization of dendritic spines [25,50,51]. An altered coordination pattern therefore cannot automatically inherit all of the efficacy accumulated during practice of the prior pattern. Connections newly required for the altered movement must acquire or stabilize efficacy through their own activity, whereas previously strengthened connections that no longer match the new coordination may become susceptible to weakening.

We propose that this weakening can reflect active interference rather than passive loss through disuse alone. Altered coordination changes the correlation, firing-rate, and timing relationships experienced by neurons within the partially overlapping ensemble. The direction and magnitude of synaptic plasticity depend jointly on relative spike timing, repetition rate, initial synaptic state, and cooperativity among inputs, and LTD can be expressed through AMPA-receptor endocytosis [50,52,53]. Connections that remain appropriately aligned with postsynaptic firing may therefore undergo Hebbian maintenance or potentiation, whereas those repeatedly recruited under decorrelated or systematically altered timing conditions may weaken or fail to retain previously stabilized efficacy. Applying these principles to an altered human TSMS remains a mechanistic extrapolation.

The same timing-based interference may affect the inhibitory motifs coupled to the excitatory ensemble. Plasticity at excitatory synapses onto inhibitory interneurons varies according to interneuron type, and inhibitory synapses onto pyramidal neurons can undergo timing-dependent potentiation or depression [28,54,55]. Altered coordination could therefore change E-E, E→I, and I→E efficacy differently. Previously useful E-E connections may weaken when their activity no longer predicts postsynaptic firing; altered pyramidal-interneuron timing may reduce the subsequent recruitment of inhibitory cells; and inhibitory output may become less effectively aligned with its pyramidal targets. The framework does not require a universal direction of plasticity at every inhibitory synapse, but proposes that reorganization of the relevant timing relationships can partially destabilize the matched excitatory and inhibitory architecture of the prior TSMS.

Several additional forms of plasticity may reinforce this redistribution. Heterosynaptic plasticity can coordinate changes across paired and unpaired excitatory and inhibitory inputs and influence the local E/I set point [29]. Recent activity can alter the threshold or direction of subsequent plasticity through metaplastic mechanisms, including changes associated with NMDA-receptor function [56]. Synaptic tagging and capture allows transiently tagged excitatory synapses to use plasticity-related products generated by stronger activity elsewhere in the same neuron [57], while motor learning can rapidly reorganize excitatory spines and interneuron boutons in M1 and selectively stabilize a subset of newly formed structures [24,51]. Together, these processes could favor stabilization of connections repeatedly aligned with the altered movement while allowing poorly aligned connections to weaken or fail to reconsolidate. Whether analogous tagging-and-capture mechanisms stabilize E→I or I→E motifs within an M1 TSMS remains unknown.

This account does not require a fixed, circuit-wide supply of potentiation that must be divided between the prior and altered patterns. Efficacy is instead proposed to be redistributed through changes in activity statistics, synapse-specific competition, and selective consolidation. Some elements of the prior TSMS remain appropriate and are preserved, whereas others weaken and newly relevant motifs remain incompletely strengthened.

We use the term partial baseline shift to describe the resulting reduction in the functional capacity of the altered TSMS. The shift is partial because overlap with the prior coordination preserves sufficient circuitry for lower-demand performance rather than eliminating the movement entirely. At higher task demands, however, the preserved and newly recruited excitatory and inhibitory motifs may not yet possess the efficacy or coordination required to reproduce the performance capacity of the previously consolidated movement.

#### 2.2.3. Movement Intensity and Functional TSMS Capacity

Within this framework, movement intensity denotes the level of task demand placed on a TSMS. Its behavioral expression is task-specific and may involve speed, force, movement amplitude, pressure, vocal intensity, or a combination of these variables. Piano performance may vary in keystroke speed and force, writing in speed and pen pressure, and phonation in loudness and articulatory demand. Movement intensity therefore does not correspond to one universal physical variable across all FTSD phenotypes.

At the neural level, increasing intensity is hypothesized to involve changes in the magnitude and temporal organization of population activity, including recruitment of additional pyramidal neurons, increases in mean firing rate, and changes in population timing or synchrony. This activity is shaped by premotor and supplementary motor regions, basal ganglia-thalamo-cortical circuits, cerebellar pathways, and sensory feedback. The present framework represents the final cortical expression of this broader network command in simplified form as the net drive delivered through the relevant TSMS.

Attempted intensity denotes the speed, force, loudness, or other task demand that the individual intends to produce, whereas realized output denotes the movement that is actually produced. Under normal conditions, the two remain appropriately matched throughout the functional range of the task.

We define the functional capacity of a TSMS as the highest task demand that its current excitatory and inhibitory architecture can implement with the intended movement pattern and without either task-specific underproduction or dystonic output. Excitatory efficacy influences how strongly incoming activity recruits and depolarizes the pyramidal population, allowing a motor command to produce larger and more reliable corticospinal output. If excitatory efficacy is reduced, further increases in attempted intensity may produce smaller-than-intended increases in descending output and lower the maximum speed or force that can be implemented normally.

Inhibitory efficacy determines whether that output remains appropriately timed, scaled, and spatially selective. E→I connections influence how effectively increasing pyramidal activity recruits rapid inhibitory interneurons, while I→E connections determine how strongly those interneurons constrain pyramidal output. When these motifs scale appropriately with excitation, task demand can increase without uncontrolled firing or recruitment of competing representations. If rapid inhibitory control is disproportionately weak, the same increase in attempted intensity may instead produce excessive, spatially diffuse, or poorly regulated activity.

The operating state of a TSMS can therefore be described through the relationship between excitatory output capacity and inhibitory control capacity. When both are sufficiently strong and proportionate, attempted intensity is translated into the intended movement throughout the normal range. When both are reduced but remain approximately proportionate, controlled performance may be preserved at lower intensities while higher-intensity output becomes insufficient. When excitation becomes excessive relative to inhibition, increasing attempted intensity may recruit a hyperexcitable regime and produce involuntary output. These latter states form the basis of the proposed true-weakness and dystonic conditions.

#### 2.2.4. Proposed True-Weakness State

We use the term true weakness to denote a proposed state of task-specific output limitation in which an attempted increase in movement intensity fails to produce the corresponding increase in speed, force, or other relevant output, despite preserved lower-intensity performance and relative preservation of strength and dexterity outside the affected task. Unlike overt dystonia, true weakness is characterized by insufficient production of the intended movement rather than excessive or involuntary output.

Within the proposed etiological sequence, true weakness follows a partial baseline shift that lowers functional TSMS capacity while preserving an approximately proportionate E/I relationship. The altered movement can therefore remain accurate at lower demands, but at higher attempted intensities the residual functional synergy lacks sufficient excitatory gain to generate the intended descending output. Increasing effort then produces a smaller-than-intended increase in realized output, while the circuit remains broadly balanced rather than entering the excitation-dominant regime proposed for overt dystonia.

We define the true-weakness threshold as the highest task intensity at which attempted intensity can still be translated faithfully into realized output by the residual functional synergy. At or below this threshold, output scales appropriately with task demand. Above it, further increases in attempted intensity generate disproportionate effort with inadequate increases in realized output. In a three-state intensity landscape, the true-weakness range lies above the normal functional range but below the intensity at which an established dystonic synergy becomes dominant.

True weakness is introduced as a testable behavioral and mechanistic construct rather than a validated clinical category. Prospective studies will need to determine whether such an intensity range can be measured reproducibly and distinguished from fatigue, pain, anxiety, peripheral neuromuscular weakness, biomechanical constraint, and compensatory movement.

#### 2.2.5. Overreaching and Excitatory-Biased Plasticity

We use the term overreaching to denote repeated recruitment of a TSMS beyond the level of activity that its current excitatory and inhibitory architecture can implement normally. Overreaching may occur directly when the affected TSMS is intentionally driven above its reduced functional capacity, or indirectly when strong activation of a neighboring TSMS generates excitatory spillover that exceeds the surround-inhibitory capacity required to keep the affected representation suppressed.

In either case, the motor command is strong enough to recruit portions of the relevant excitatory ensemble but the weakened circuit cannot generate or regulate the complete population pattern required for the intended motor state. Activity may therefore become fragmented, weakly synchronized, temporally inconsistent, or insufficiently constrained. During direct overreaching, this produces an incomplete attempt to generate the intended high-intensity movement; during indirect overreaching, it produces partial or inappropriate recruitment of a TSMS that should have remained suppressed.

The central hypothesis is that repeated activity in this fragmented regime biases plasticity toward recurrent excitation. The relevant activity need not eliminate inhibitory recruitment. Rather, it must be sufficient to strengthen at least a subset of E-E connections while being less favorable for the matched strengthening of E→I and I→E circuitry.

Several properties of cortical plasticity make such an asymmetry plausible. First, induction rules differ across synapse classes. Plasticity at excitatory cortical synapses depends jointly on spike timing, repetition rate, initial strength, and cooperativity, whereas E→I plasticity varies according to the postsynaptic interneuron type [52,53,54]. A fragmented activity pattern could therefore affect E-E connections and excitatory recruitment of inhibitory neurons differently.

Second, rapid feed-forward and somatic inhibition narrows the temporal integration window of pyramidal neurons and improves spike-time fidelity [58,59]. Weakly synchronized or temporally dispersed excitatory packets may consequently recruit rapid inhibitory control less consistently than coherent population activity. The framework predicts that such activity can remain sufficient for incremental E-E strengthening while failing to produce equally reliable E→I adaptation.

Third, I→E synapses are themselves plastic. Timing-dependent changes have been demonstrated at cortical inhibitory synapses, including PV-interneuron output synapses, although their direction and induction requirements depend on timing, circuit context, and the pre-existing E/I state [28,55]. Irregular interneuron recruitment during overreaching may therefore fail to generate the temporal relationships needed for strengthening of inhibitory output onto the active pyramidal population.

Finally, consolidation mechanisms could amplify an initially small asymmetry. Synaptic tagging and capture allows transiently potentiated excitatory inputs to use plasticity-related products generated by stronger activity elsewhere in the same neuron [57]. Active E-E connections may therefore be tagged and stabilized more consistently than inhibitory motifs that are recruited weakly or irregularly. The extension of this mechanism to the differential stabilization of E-E, E→I, and I→E circuitry in an M1 TSMS remains speculative.

These mechanisms are not mutually exclusive, and the proposed sequence has not been demonstrated directly in FTSD. Together, they define a testable plasticity hypothesis: repeated overreaching generates an activity regime that strengthens a recurrent excitatory subnetwork without producing proportionate strengthening of its rapid inhibitory control.

#### 2.2.6. Formation of the Dystonic Synergy and the Symptom-Threshold

Under the proposed pathway, repeated excitatory-biased plasticity may gradually produce a functionally differentiated subnetwork in which recurrent excitation has strengthened beyond the capacity of its matched rapid inhibitory control. We refer to this excitatory-dominant configuration as a dystonic synergy. It is not assumed to be anatomically separate from the residual functional synergy; instead, the two configurations may contain partially overlapping neurons and synapses while differing in their effective connectivity, recruitment conditions, and predominant operating ranges.

During its early formation, the dystonic synergy may function as a high-intensity extension of the residual functional synergy. Continued overreaching is hypothesized to increase the probability that an excitatory subset will recruit itself through recurrent activity. Once task-related input reaches a range in which this configuration is more readily recruited than the residual functional configuration, inadequate inhibitory control permits excessive, spatially inappropriate, or poorly regulated pyramidal activity, producing involuntary contraction, abnormal posturing, or competing motor output.

The behavioral symptom-threshold marks this change in relative dominance. At or below the threshold, the residual functional synergy retains control of voluntary, symptom-free movement; above it, the dystonic configuration becomes preferentially recruited. The symptom-threshold therefore represents a crossover between functional network states rather than the absence of dystonia-related circuitry at lower intensities.

Because the functional and dystonic configurations may remain partially overlapping, some pyramidal neurons and inhibitory elements may participate in both lower-intensity voluntary movement and the higher-intensity dystonic state. Repeated higher-intensity recruitment, together with the metaplastic, synaptic-consolidation, and structural-remodeling processes discussed above [51,56,57], could progressively stabilize the dystonic configuration while the residual functional synergy continues to govern lower-demand performance.

This progression has not been observed directly at the circuit level. It provides a candidate explanation for the coexistence of normal lower-intensity performance and abnormal higher-intensity output and generates a specific prediction: experimentally increasing inhibitory control relative to recurrent excitation should raise the symptom-threshold, whereas weakening that control should lower it.

#### 2.2.7. Persistence and a Three-State Intensity Landscape

Once repeatedly consolidated, a dystonic configuration may become relatively stable without becoming anatomically independent of the residual functional synergy or immune to later plasticity. Persistence may partly reflect divergent activation histories: lower-intensity, symptom-free performance continues to recruit and reshape the residual functional configuration, whereas the dystonic configuration is recruited mainly at higher intensities and may be engaged less often when symptom-provoking movements are avoided. A new coordination pattern practiced below the symptom-threshold may therefore modify the functional configuration more strongly than the dystonic one, allowing relative preservation of the latter through differential recruitment rather than complete circuit separation.

Saturation or occlusion of excitatory plasticity may also stabilize the dystonic configuration without requiring indefinite progression. Motor-skill learning can strengthen horizontal connections in M1 while reducing the capacity for further experimentally induced LTP [26,27]. If repeated overreaching brings a substantial portion of the dystonic configuration’s E-E connectivity toward a local potentiation ceiling, subsequent recruitment may produce diminishing additional strengthening, allowing the abnormal pattern to persist without continually increasing in severity.

If residual functional capacity declines further while the established dystonic configuration remains recruitable at higher task demands, a three-state intensity landscape may emerge. At or below the true-weakness threshold, attempted intensity remains faithfully translated into realized output. Between the true-weakness threshold and the symptom-threshold, task demand exceeds functional capacity without yet preferentially recruiting the dystonic configuration, producing true weakness. Above the symptom-threshold, the dystonic configuration becomes dominant and overt symptoms emerge. This organization is predicted only when a measurable interval separates the two thresholds, potentially following further changes in movement coordination without adequate consolidation. These boundaries also define the intact range targeted by BATR.

### 2.3. Rehabilitative Hypothesis: Below- or At-Threshold Retraining

#### 2.3.1. Definition and Relationship to Existing Retraining Approaches

Within the present framework, the central rehabilitative prediction is that repeated practice confined to a symptom-free and functionally intact range may progressively rebalance activity within the affected TSMS. We refer to this principle as below- or at-threshold retraining (BATR). BATR is defined as systematic practice of the affected movement at or below the highest task intensity at which it can be executed accurately and without overt dystonic symptoms.

In a proposed three-state intensity landscape that also includes true weakness, the relevant training limit is the lower of the symptom-threshold and the true-weakness threshold. Practice above the true-weakness threshold, even if it remains below the symptom-threshold, would exceed the current capacity of the residual functional synergy and could reproduce the fragmented activity regime hypothesized to promote maladaptive plasticity. BATR therefore confines practice to a range in which attempted intensity and realized output remain appropriately matched.

The variable defining this range is task-specific and need not be movement speed alone. Depending on the FTSD phenotype, the relevant demand may include force, movement amplitude, pressure, vocal intensity, intereffector coordination, or a multidimensional combination of variables. Practice should recruit the intended movement reliably while remaining within the current functional capacity of the TSMS. The optimal location within this range, the required number of repetitions, and the most effective progression schedule remain the subjects of empirical questions.

BATR is conceptually related to the slow-down exercise described by Sakai [15] and subsequently examined by Yoshie et al. [17], as well as to related sensorimotor retraining approaches evaluated in musicians with dystonia [16]. These reports provide clinical precedent for the possibility that carefully constrained, symptom-sparing practice can improve task performance, but they do not establish a universal treatment protocol or demonstrate that improvement occurs through PV-mediated synaptic strengthening.

The principal contribution of BATR is therefore not simply a recommendation to perform the task slowly. It defines practice relative to an individualized symptom-threshold and, where applicable, a true-weakness threshold; allows the controlling task variable to differ across FTSD phenotypes; and links symptom-free performance to a specific, testable hypothesis of TSMS-level E/I rebalancing.

#### 2.3.2. Proposed Neurophysiological Mechanism of BATR

Whether BATR improves task performance and whether any resulting improvement is mediated by TSMS-level E/I rebalancing are separate empirical questions. The proposed mechanism of BATR follows from the contrast between activity within and beyond the functional capacity of a TSMS. During overreaching, pyramidal activity is hypothesized to become fragmented, weakly coordinated, or insufficiently constrained, favoring recurrent excitatory strengthening without proportionate adaptation of rapid inhibitory control. During BATR, by contrast, practice remains within a range that the residual functional synergy can implement normally. Pyramidal activity is therefore predicted to be more coherent and to recruit matched inhibitory circuitry more reliably.

Repeated high-fidelity performance within this range may promote coordinated adaptation across E-E, E→I, and I→E motifs. Motor learning in M1 remodels both excitatory and inhibitory circuitry, although these changes are phase- and subtype-specific rather than a simple monotonic increase in inhibition. Learning has been associated with increased PV-related perisomatic boutons, reduced SST-related distal boutons, and dynamic changes in glutamatergic and GABAergic transmission [24,25]. In other cortical preparations, coordinated homosynaptic and heterosynaptic plasticity across excitatory and inhibitory inputs can adjust the local E/I relationship [28,29]. On this basis, we hypothesize that symptom-free practice produces activity patterns that recruit rapid inhibitory circuitry more consistently and support more proportionate adaptation of excitatory and inhibitory motifs than above-capacity practice.

The framework further proposes that the functional and dystonic configurations are only partially segregated and may share pyramidal neurons, inhibitory interneurons, and local inhibitory projections. If PV interneurons recruited during functional movement also receive input from, or project onto, pyramidal neurons participating in the dystonic configuration, repeated symptom-free recruitment could strengthen E→I input onto shared inhibitory neurons and I→E output across a broader local pyramidal population. BATR could therefore improve inhibitory control of both configurations without repeatedly driving the excitatory-dominant dystonic state into overt expression.

Because some pyramidal neurons may participate in both configurations, dystonia-related circuitry need not be completely silent during below-threshold movement. The critical distinction is which configuration organizes population activity. During BATR, the residual functional configuration is predicted to remain dominant and recruit rapid inhibition effectively, favoring balanced circuit stabilization rather than further consolidation of the dystonic state.

As rapid inhibitory control strengthens relative to recurrent excitation within the dystonic configuration, progressively greater task-related input should be required to recruit the hyperexcitable state. The principal behavioral prediction is therefore an upward shift of the symptom-threshold, with previously dystonia-provoking intensities remaining under functional control. In a three-state landscape, strengthening of the residual functional synergy could also raise the true-weakness threshold by expanding the range over which attempted intensity is translated accurately into realized output. This recovery need not require immediate erasure of the excitatory connections supporting the dystonic configuration. Functional rebalancing may occur once inhibitory recruitment and output become sufficient to constrain those connections across the required range of task demand, although longer-term practice could additionally remodel maladaptive excitatory relationships.

If BATR operates through the proposed mechanism, behavioral improvement should covary with the recovery of rapid intracortical inhibition, surround inhibition, and related physiological measures of motor selectivity. Section 4.3.1 develops the corresponding support and challenge criteria. BATR should therefore be regarded as a hypothesis-driven rehabilitation strategy rather than an established treatment. Its efficacy, safety, durability, optimal dosage, and generalizability require prospective clinical evaluation.

### 2.4. Materials and Methods

Computational modeling was used as a complementary proof-of-concept approach to examine a restricted network-level implication of the proposed TSMS framework. Specifically, we asked whether a simplified spiking neural network containing functional and excitation–inhibition (E/I)-imbalanced components could exhibit an input-dependent crossover in relative population firing. The model was not designed to reproduce the complete proposed etiological sequence from altered movement coordination through true weakness, overreaching, and formation of a dystonic synergy. It also does not simulate muscular output, clinical symptoms, or recovery through BATR.

Instead, the simulation examines the conditional behavior of a specified network architecture in which the functional and E/I-imbalanced states are represented directly. Because the relevant imbalance is imposed within the model, the results address the internal computational plausibility and network-level consequences of that assumption rather than independently verifying its biological origin or establishing it as the mechanism of human FTSD.

#### 2.4.1. Network Model

We simulated a simplified spiking neural network composed of interconnected excitatory and inhibitory components. The network contained *N*_ex_ excitatory neurons and *N*_inh_ inhibitory neurons governed by a leaky integrate-and-fire (LIF) model with synaptic interactions. The model was deliberately reduced to isolate the relationship between E/I organization, external drive, and population firing. It does not reproduce the full cellular diversity, laminar organization, long-range connectivity, sensory feedback, or descending motor-output pathways of an M1 TSMS. Although the biological hypothesis emphasizes PV-centered inhibitory control, the inhibitory units in the simulation are generic model neurons and should not be interpreted as a cell-type-specific representation of PV interneurons.

The neurons evolved according to the following membrane potential equation:

$$\tau_{m}dV/dt\;=\;(V_{rest}-V)\;+\;I_{syn}$$

where *V* is the membrane potential, *V*_rest_ is the resting potential, *τ*_m_ is the membrane time constant, and *I*_syn_ represents synaptic input.

Spiking events were generated when *V* exceeded a threshold *V*th, after which the membrane potential was reset to *V*_reset_.

#### 2.4.2. Synaptic Connectivity

Synaptic interactions were implemented with conductance-based synapses, where synaptic current was given by the following:

$$I_{syn}=g_{E}s(V-E_{E})+g_{I}s(V-E_{I})$$

where *g*_E_ and *g*_I_ are the excitatory and inhibitory synaptic conductance, *E*_E_ and *E*_i_ are the excitatory and inhibitory reversal potential of the synapse. Excitatory-to-inhibitory (E→I) and inhibitory-to-excitatory (I→E) connections were defined with a probability of connection *p*. Upon a synaptic input, the strength of the communication is increased by *s* after an axonal delay of 1.5 ms, and later it decays exponentially.

The modeled connection patterns and synaptic strengths represent specified network conditions. They are not intended to reproduce all synaptic motifs of a biological M1 microcircuit or to identify a particular synaptic abnormality in patients with FTSD.

#### 2.4.3. Input Drive and External Stimuli

External input was applied to the neurons through a Poisson-distributed spike train representing an abstract combination of background activity and task-related drive. Input amplitude to the excitatory populations was varied systematically to characterize how the modeled populations responded as drive increased. Within the conceptual interpretation of the model, increasing drive corresponds qualitatively to increasing task demand. It is not, however, calibrated directly to movement speed, force, loudness, or any other behavioral or clinical measure.

The external drive followed

$$I_{ext\;}=A\times Poisson(r)$$

where *A* denotes the input amplitude and *r* denotes the baseline firing rate of the Poisson process.

#### 2.4.4. Representation of Functional and E/I-Imbalanced Synergies

To represent the two modeled network configurations, we specified two model populations: a functional synergy population (FS) and a dystonic synergy population (DS). The FS population was assigned balanced excitatory and inhibitory drive, represented by *E*_F_ and *I*_F_, whereas the DS population was assigned relatively increased excitatory strength without a corresponding increase in inhibitory strength, represented by *E*_D_ and *I*_D_. FS and DS are model constructs rather than directly recorded biological populations.

The E/I imbalance was imposed rather than generated through simulated movement reorganization, overreaching, or synaptic plasticity. Systematically increasing model drive therefore tests how the FS and DS populations respond under their respective E/I conditions as model demand increases. It should not be interpreted as a simulation of the biological formation of dystonia or as evidence that overreaching produces the imposed synaptic imbalance in human FTSD.

#### 2.4.5. Firing Rate Analysis

Network activity was summarized using the population-averaged firing rates of the FS and DS populations. Firing rate was calculated as follows:

$$r=\frac{total\;spikes}{number\;of\;neurons\;\times simulation\;time}$$

For each tested input-strength value, the population-averaged firing rates of the FS and DS populations were calculated and plotted against input strength.

#### 2.4.6. Simulation and Implementation

All simulations were implemented in Python 3.10.13 using custom-built neuron models. Differential equations were integrated using the Euler method with a time step of *dt* = 0.1 ms. Simulations were run with different seeds to account for variability. The network was simulated for *T* = 2000 ms, and simulation outputs were processed and summarized descriptively using Pandas and visualized with Matplotlib 3.8.2. In Table 2, we show parameters implemented in the simulation code, covering single-neuron dynamics, synaptic properties, network structure, external drive, and numerical integration. Values correspond to the PYR class defaults and network/simulation parameter block used to generate the reported results.

#### 2.4.7. Robustness and Sensitivity Analyses

Because the model is reduced and several of its parameters are not constrained by measurement, we evaluated how far the reported population-level result depends on the specific realization, network size, and parameter values chosen. Four analyses were performed.

**Stochastic and structural replication**. The full input-strength sweep (s = 0.5 to 3.0, eleven values) was repeated with 40 independent random seeds. Because the manuscript’s conceptual description of the DS population does not uniquely determine its connectivity, the sweep was run under four structural variants: (V1) DS with recurrent excitation and no inhibition, external drive amplitude specified independently of synaptic weight; (V2) as V1, but with the DS population also receiving inhibition from the FS interneuron pool; (V3) DS without recurrent connections, receiving external drive only; and (V4) as V1, but with the external drive amplitude of each population set equal to that population’s own excitatory synaptic weight. For each seed and variant, we computed the difference in population-averaged firing rate, DS − FS, at every input strength, and identified the input strength at which this difference first changed sign from negative to positive by linear interpolation.

**Network size.** The sweep was repeated for excitatory population sizes of N_E_ = 10, 20, 40 and 80, with the interneuron count held at N_E_/4, using 15 seeds per size. Two weight conventions were compared, and synaptic weights scaled in proportion to 1/Npre so that total synaptic drive per cell is invariant to network size.

**One-at-a-time (OAT) sensitivity.** Each of nine parameters was perturbed by ±20% from baseline with all others held fixed, and the network re-simulated at three operating points (s = 1.0, 2.0, and 3.0) rather than at a single drive level. Each perturbation was replicated across 30 seeds, and effects are reported with 95% intervals across seeds. Sensitivity was quantified as the absolute change, in Hz, of the difference DS − FS. This choice is deliberate: the percentage change is undefined when a population is quiescent and uninformative when a population is near the refractory ceiling, whereas the absolute change in DS − FS is defined in both cases, uses the same units for both populations, and is the quantity on which the population comparison rests. The spike threshold was perturbed as the rest-to-threshold distance (Vth − EL), because Vth is defined on an interval scale and a multiplicative perturbation of −50 mV is not interpretable (a +20% change would place the threshold at −60 mV, equal to Vreset).

**Global sensitivity**. Because OAT analysis cannot detect parameter interactions, we additionally drew 400 Latin hypercube samples from a ±20% box around the baseline values of all nine parameters simultaneously, and evaluated the network at s = 1.0, 2.0, and 3.0. Parameter influence was summarized using standardized regression coefficients (SRCs) on DS − FS, with the coefficient of determination reported as an index of how much of the response is captured by a linear additive model. We also report the proportion of sampled parameter sets in which DS exceeded FS, as a direct test of whether the qualitative ordering survives simultaneous variation in parameters.

All simulations used the same integration scheme, time step and duration as the main analysis. The refractory ceiling implied by τref = 2.0 ms, namely 1/τref = 500 Hz, is shown alongside firing rates where relevant so that saturation is visible.

## 3. Results

The simulations characterized the firing behavior of the FS and DS model populations. We first examined activity under the functional reference condition, then considered alternative input–output behavior under different E/I conditions, and finally compared the relative firing rates of the FS and DS populations. These conditions should be understood as illustrative network states rather than as successive stages in the biological development of dystonia.

Figure 1B presents a representative raster plot from the functional reference condition. Under the selected parameterization, the network displayed stable spiking activity over the simulated interval.

Figure 1C shows the relationship between input synaptic strength and population firing rate in the same model condition. Across the tested range, firing rate increased as input synaptic strength increased. The inset shows the corresponding activity of the inhibitory model units.

The relationship between increasing external drive and population firing was not identical across all specified network conditions. In an E/I network, stronger external input can increase excitatory firing while simultaneously recruiting greater inhibitory feedback through the network’s connectivity. Depending on the imposed relationship between excitation and inhibition, this feedback may limit, stabilize, or reduce the net population response as input increases.

Figure 1D illustrates an alternative input–output pattern in which firing rate decreases across increasing input strength under the selected model parameters.

Together, Figure 1C,D demonstrate that the simplified model can produce qualitatively different input–output relationships under different specified E/I conditions.

Figure 1E compares the population-averaged firing rates of the FS and DS populations across increasing input strength. At lower values, DS firing remained below FS firing. Above the model-level crossover, this ordering reversed and DS exhibited the higher firing rate. The imposed difference in E/I organization therefore produced an input-dependent crossover between the two firing-rate curves.

This result supports a restricted conditional proposition: under the specified architecture and parameterization, different E/I configurations can change their relative population firing as model drive increases. The crossover is interpreted as an abstract network-level analogue of threshold-dependent recruitment; it does not independently establish biological competition between motor synergies or simulate involuntary contraction, muscular output, maladaptive plasticity, or clinical FTSD.

### 3.1. The Population Difference Depends on Unstated Structural Choices

Across 40 seeds, the qualitative relationship between the FS and DS populations also differed by structural variant rather than being a property of the imposed E/I imbalance (Figure 2). This is not surprising, as recurrent networks can achieve the same net input in many ways, a phenomenon known as degeneracy. Under variants V1 and V2, in which the DS population carries recurrent excitation and the external drive amplitude is specified independently of synaptic weight, the DS population exceeded the FS population at every input strength tested, including the lowest (40 of 40 seeds in both variants); no reversal of ordering occurred anywhere in the range. Under variant V3, in which the DS population receives external drive only, the two populations were statistically indistinguishable, with the 95% interval of DS − FS spanning zero at every input strength. Under variant V4, in which drive amplitude scales with synaptic weight, the DS population rose from quiescence to a plateau between s = 1.25 and s = 2.0 and the ordering reversed in all 40 seeds at s = 1.16 ± 0.19.

The two variants that produce a reversal of ordering are therefore V4, whose defining feature is that the parameter labelled as the DS excitatory weight also sets the DS external drive, and V3, in which the apparent reversal is not distinguishable from Poisson variability. We conclude that the reversal is a consequence of specific structural choices rather than a robust consequence of E/I imbalance itself.

### 3.2. Both Populations Operate Outside a Usable Dynamic Range

In variants V1 and V2 the DS population fired at 402–467 Hz across the whole input range, that is at 80–93% of the 500 Hz refractory ceiling, while the FS population remained flat near 4.5 Hz (V1) or near 0.1 Hz (V2). In variant V4 the FS population did not spike at any input strength (0.00 Hz throughout) while the DS population moved from 0 Hz to 466 Hz. In neither case is there a graded input–output relationship available for comparison: the difference between the populations reflects one population sitting at a floor and the other at a ceiling. This also means that percentage change metrics computed at these operating points are not interpretable, as the denominator is either zero or already at saturation.

### 3.3. The Transition Point Is Not Invariant to Network Size

Under variant V4, the input strength at which the ordering reverses depended on network size, and the direction of that dependence depended on the weight convention (Figure 3). With synaptic weights held at their Table 2 values, the transition shifted modestly from s = 1.22 ± 0.25 at N_E = 10 to s = 0.89 ± 0.11 at N_E = 80, although in this convention total synaptic drive per cell grows with network size, so the four networks are not equivalent models. Under the size-invariant convention in which weights scale as 1/N_pre, the transition moved by more than a factor of four, from s = 0.50 at N_E = 10 to s = 2.11 ± 0.22 at N_E = 40, and no reversal occurred at all at N_E = 80 (0 of 15 seeds). The location of the transition is therefore a property of the particular network size chosen and not a scale-invariant feature of the model.

### 3.4. Local Sensitivity Around a Non-Saturated Operating Point

Because sensitivity analysis is uninformative at a floor or a ceiling, we searched for a parameter regime in which both populations spike at physiologically plausible rates. Of 108 combinations of the DS excitatory weight, the FS inhibitory weight and the external drive amplitude, 25 left the DS population above 300 Hz, 85 placed the DS population above the FS population at every input strength, and one combination produced both populations spiking below 20 Hz together with a reversal of ordering within the tested range. OAT sensitivity was evaluated in that regime (Figure 4).

At s = 1.0 and s = 2.0, where both populations fire in the 10–15 Hz range, all nine parameters produced effects on DS − FS of at most 5 Hz, with membrane capacitance, external drive amplitude, synaptic decay time constant and rest-to-threshold distance producing the largest changes and the FS synaptic weights producing the smallest. At s = 3.0, however, a −20% change in the rest-to-threshold distance increased DS − FS by 193 Hz (95% interval 118 to 225 Hz), that is it moved the DS population from approximately 15 Hz into saturation. The regime is therefore stable to parameter perturbation at moderate drive but sits within approximately 20% of a bifurcation into runaway excitation at the highest drive tested.

### 3.5. Global Sensitivity and Survival of the Qualitative Result

Varying all nine parameters simultaneously within a ±20% box (400 Latin hypercube samples, variant V4) separated the robust and non-robust components of the result (Figure 5). The ordering DS > FS held in 100% of parameter sets at s = 3.0 and in 97% at s = 2.0, but in only 18% at s = 1.0. In other words the existence of a difference at high drive is robust to simultaneous parameter variation, whereas the drive level at which that difference appears is not: at s = 1.0 a ±20% perturbation reverses the result in more than four of five parameter sets.

Standardized regression coefficients identified the rest-to-threshold distance (SRC = −0.45), the DS excitatory weight (+0.44) and the synaptic decay time constant (+0.28) as the leading contributors at s = 2.0. However, the linear additive model accounted for only R^2^ = 0.13 to 0.56 of the variance in DS − FS across the three operating points. This low explanatory power is itself informative: the response is threshold-like rather than approximately linear in the parameters, which is precisely the situation in which a local, one-at-a-time analysis is least able to characterize model behavior. We therefore report the global analysis alongside, rather than instead of, the OAT result.

### 3.6. What Survives in a Non-Saturated Regime

Replicating the single non-saturated regime across 40 seeds (Figure 6), the FS population was flat across the entire input range (10.27 ± 0.67 Hz at s = 0.5; 10.29 ± 0.70 Hz at s = 3.0) while the DS population increased from 10.35 ± 0.78 Hz to 15.78 ± 0.90 Hz. The separation at high drive was large relative to between-seed variability (DS − FS = 5.49 ± 1.11 Hz at s = 3.0; standardized effect size 4.9) and DS exceeded FS in 40 of 40 seeds for every input strength at or above s = 1.5.

The ordering at low drive was not reliable. At s = 0.5 the DS population exceeded the FS population in 45% of seeds, and a reversal of ordering within the tested range was identified in only 26 of 40 seeds; in the remaining 14 the DS population was already above the FS population at the lowest input strength tested. Even under the most favorable configuration we could identify, therefore, the FS population is not reliably above the DS population at low drive, and the model does not support the identification of a transition point only due to E/I balance, but also could show up via structural changes, a proposition that deserves more attention in later experiments.

## 4. Discussion

### 4.1. Relation to Existing Models and Literature

A central question is how the M1-centered TSMS hypothesis relates to basal ganglia-, cerebellar-, and distributed-network accounts of FTSD. Basal ganglia models emphasize the contribution of cortico-striato-pallido-thalamo-cortical pathways to facilitation of intended movements and suppression of competing output. Altered direct- and indirect-pathway signaling may disinhibit thalamocortical activity and contribute to abnormal motor cortical excitability [8,60,61,62,63,64,65]. Cerebellar accounts emphasize abnormal motor calibration, sensorimotor integration, and cerebello-cortical plasticity [66,67,68], whereas distributed-network models conceptualize FTSD as emerging from abnormal interactions among cortical, basal ganglia, thalamic, cerebellar, and sensory systems without assigning exclusive causal primacy to one node [69,70,71].

These perspectives operate at partly different explanatory levels and need not be mutually exclusive. The distinctive claim of the TSMS framework concerns the proposed initiating locus and causal ordering: the decisive E/I imbalance is hypothesized to arise within an M1 ensemble that directly contributes to the affected movement component, while abnormalities elsewhere in the motor network shape, reinforce, or adapt to its expression.

Locating the proposed initiating imbalance within a task-dependent M1 ensemble offers a candidate account of task-restricted, intensity-dependent symptom expression, conditional on recruitment of that ensemble in the affected task context. Maladaptive plasticity is hypothesized to be concentrated within the ensemble recruited by the affected movement rather than distributed uniformly across the motor system. At lower task demands, the residual functional configuration is proposed to retain control; above the symptom-threshold, the excitatory-dominant dystonic configuration becomes preferentially recruited. The process by which task context selects the relevant ensemble is assumed rather than explained by this account.

This conditional account does not establish M1 primacy. Basal ganglia, cerebellar, premotor, and broader network processes can also be organized somatotopically and according to task context. A distributed or subcortical-primary model could therefore accommodate narrowly expressed symptoms if the affected network channel were sufficiently task-specific. The TSMS proposal should be evaluated not simply by whether it can accommodate task-restricted symptoms, but by whether its predictions concerning causal timing, cell-type-specific physiology, and threshold-dependent recruitment are supported more strongly than those of competing accounts.

Prior computational work has addressed dystonia at different anatomical levels and has targeted different physiological or behavioral phenomena. Sanger and Merzenich [72] tested whether altered sensory representations could generate abnormal motor behavior in FTSD. Sanger [73], in a model of childhood-onset generalized dystonia, showed that excessive gain in an indirect-pathway basal-ganglia feedback loop could produce unstable synchronous activity in cortex and basal ganglia. Sohn et al. [74] used a neuromorphic sensorimotor network to test whether correlated sensory input and spike-timing-dependent plasticity could produce persistent motor overflow in focal hand dystonia. Neymotin et al. [75] used a biologically detailed, multiscale M1 model to reproduce dystonia-like cortical hyperactivity and beta synchrony and to explore multitarget pharmacological transitions toward physiological dynamics. Geminiani et al. [76] used a data-driven cerebellar spiking network to test whether specific cerebellar alterations could reproduce dystonia-related deficits in associative motor learning.

These models instantiate distinct, and in some cases competing, causal assumptions. Their results establish conditional consequences within their specified architectures; they do not independently establish that the modeled abnormality is the initiating mechanism of the corresponding human dystonia phenotype. The present model asks a narrower question: given directly specified functional and E/I-imbalanced population states, can increasing abstract drive reverse their relative firing-rate ordering? The comparison therefore concerns explanatory level, target phenotype, and model scope rather than etiological agreement.

The reported responsiveness of some FTSD cases to slowdown exercise and related sensorimotor retraining is compatible with a plasticity-based mechanism but is not uniquely diagnostic of an M1 TSMS abnormality [15,16,17]. Improvement could arise through changes in M1, sensory processing, cerebellar error processing, compensatory recruitment of other motor representations, or broader network reorganization. Behavioral recovery therefore supports the potential reversibility of FTSD but does not by itself establish the anatomical or cellular mechanism of that recovery.

The framework further predicts that repeated activation of a hyperexcitable M1 synergy can induce or reinforce changes throughout the motor hierarchy, including altered basal ganglia signaling, cerebellar adaptation, somatosensory reorganization, and diminished spinal inhibition. These proposed network adaptations are developed in greater detail in the companion hypothesis article. Alternative causal sequences remain viable: basal ganglia or cerebellar dysfunction could precede and induce M1 disinhibition, abnormalities could arise concurrently across several nodes, or causal ordering could differ among FTSD phenotypes. Longitudinal measurements and temporally resolved interventions will be required to distinguish these possibilities.

The TSMS framework is therefore not intended to deny the distributed organization of FTSD. It proposes a hierarchy within that distributed system in which a task-specific cortical ensemble contains the initiating E/I imbalance, while the broader motor network contributes to its expression, adaptation, and persistence. Its value will depend on whether it produces discriminating predictions rather than merely accommodating findings that can be explained equally well by competing models.

### 4.2. Evidentiary Status and Limitations

The present framework is mechanistic and integrative, but several of its central constructs remain unobserved directly. The TSMS is defined as a task-dependent M1 ensemble supporting a particular unidirectional movement component. Although partially overlapping motor cortical populations are consistent with contemporary accounts of M1 organization [77,78], an FTSD-specific TSMS has not been isolated anatomically or followed longitudinally during the proposed transition from normal function to dystonia. It should therefore be understood as a functional circuit hypothesis rather than an established discrete cortical module.

Task-context selection remains an assumed upstream process in the TSMS framework, and the mechanism linking task context to recruitment of the relevant M1 ensemble is not specified. The computational model examines responses to varying abstract input drives in predefined FS and DS populations; it does not represent different task contexts or simulate context-dependent ensemble selection. It therefore does not explain task specificity.

The proposed predominance of PV-centered inhibitory dysfunction is likewise inferential. Reduced SICI, disturbed surround inhibition, and abnormal cortical excitability support dysfunction of rapid intracortical inhibition, but human TMS cannot identify the responsible interneuron class or distinguish directly between E→I and I→E synaptic abnormalities. SST- and 5HT3aR-related pathways may also contribute, and clinical phenomenology cannot establish cellular identity. Testing the PV-centered account will require the recording or manipulation of identified interneuron populations within task-relevant motor ensembles.

The etiological sequence from altered movement coordination through partial baseline shift, true weakness, overreaching, excitatory-biased plasticity, and formation of a dystonic synergy has not been observed longitudinally at the circuit level. Its component mechanisms are drawn from broader studies of motor learning and synaptic plasticity, but their proposed combination and direction within an M1 TSMS remain to be demonstrated. True weakness likewise remains a proposed construct whose validity and generality require reproducible measurement and exclusion of alternative causes of task-specific underproduction.

The causal primacy assigned to M1 also remains unresolved. Basal ganglia, cerebellar, sensory, and spinal abnormalities are well documented in dystonia, but existing cross-sectional evidence generally cannot determine whether they are causes, consequences, compensations, or interacting components of the same pathological process. The framework predicts that at least some of these abnormalities develop secondarily to repeated abnormal M1 outputs; consistent evidence that extra-M1 abnormalities precede cortical disinhibition or independently predict symptom emergence would instead favor an upstream or distributed causal account.

Evidence supporting symptom-sparing retraining remains limited to individual cases and relatively small cohorts and does not establish BATR efficacy, optimal dose, durability, safety, or generalizability [15,16,17]. Behavioral improvement alone would also not establish the proposed TSMS-level E/I rebalancing mechanism, which requires controlled clinical evaluation and longitudinal physiological testing.

The computational model addresses only the restricted proposition that specified FS and DS conditions can exhibit an input-dependent crossover in relative population firing. Because the E/I configurations and network architecture are imposed, the simulation does not establish that the modeled conditions exist biologically, arise through the proposed etiological sequence, or are reversed through BATR. Its role is to clarify one conditional network consequence of the framework and generate quantities that can guide subsequent experimental tests.

Finally, FTSD is clinically and physiologically heterogeneous. Symptom-thresholds may depend on several interacting task variables, differ across individuals and phenotypes, and appear as gradual or context-dependent transitions rather than a single abrupt boundary. The relative contributions of M1, other motor-network nodes, genetic susceptibility, sensory processing, and behavioral history may likewise vary. The framework should therefore be evaluated as a candidate causal account whose applicability may differ among FTSD phenotypes rather than as a universal mechanism established for every case.

These limitations define the observations needed to adjudicate the hypothesis. The framework will be strengthened by prospective findings that are not merely compatible with it, but that distinguish its proposed causal sequence and cellular mechanism from viable basal ganglia-primary, cerebellar-primary, and distributed-network alternatives.

### 4.3. Testable Predictions and Falsification Criteria

The central predictions of the TSMS framework can be evaluated through human longitudinal studies and experimental models. Because measures such as SICI are indirect and reflect several circuit processes, an isolated negative result would not necessarily reject the complete framework. Repeated failure of its central predictions across appropriately designed studies would, however, challenge the corresponding components of the hypothesis.

#### 4.3.1. Human Longitudinal Tests of the Symptom-Threshold and BATR

The most immediate human prediction is that, if BATR is effective, the symptom-threshold and, where measurable, the true-weakness threshold will increase. Prospective studies should compare these thresholds before and after a prespecified BATR block, with formal assessments conducted separately from routine practice. During the intervention, change could be monitored through structured participant reports and passive task-performance measures. Candidate procedures for threshold estimation, reliability analysis, and objective outcome measurement are detailed in Section 4.4.

If BATR operates through the recovery of rapid inhibitory control, an upward shift of the symptom-threshold should covary with normalization of SICI, surround inhibition, and related physiological measures of motor selectivity [5,6,32,41]. Such covariation would support the recovery of fast cortical inhibition but would not by itself establish PV-specific synaptic strengthening. Conversely, substantial behavioral improvement without corresponding recovery of these inhibitory measures would challenge the proposed PV-centered mechanism, although it would not necessarily invalidate BATR as a rehabilitation strategy. Alternative mechanisms could include sensory learning, altered attention, compensatory recruitment, or broader network reorganization.

The proposed true-weakness state can be tested by varying task intensity systematically while measuring attempted and realized output independently. The framework predicts that at least some individuals will display an interval in which increasing effort fails to produce the intended increase in output before overt dystonic activity becomes dominant. Failure to identify such an interval across carefully characterized cohorts would limit the generality of true weakness and suggest that it applies only to a subset of FTSD phenotypes or etiological pathways.

#### 4.3.2. Testing Causal Ordering Across the Motor Network

The hypothesis that M1 contains the initiating maladaptation generates specific predictions about temporal ordering. Longitudinal studies should combine M1 physiology with task-related measures of basal ganglia function, cerebello-cortical connectivity, somatosensory processing, and spinal inhibition before and during symptom change. The central prediction is that changes in the affected TSMS and its rapid inhibitory physiology will precede or closely track normalization elsewhere in the motor network.

Evidence that basal ganglia, cerebellar, sensory, or spinal abnormalities consistently precede cortical disinhibition and predict later symptom development would favor an upstream or distributed causal sequence. Earlier or independent normalization of extra-M1 measures during recovery would likewise weaken a strictly downstream interpretation, whereas simultaneous changes across several nodes could indicate that the initiating pathology cannot be localized to a single component of the network.

The timing of intervention-related changes may be especially informative. If BATR first alters M1 inhibitory physiology and later produces normalization of extra-M1 measures, that sequence would support the proposed hierarchy. If the earliest changes occur consistently in cerebellar, basal ganglia, sensory, or compensatory networks, the causal account would require revision. Temporal ordering alone would not prove causality, but it would substantially constrain the range of viable models.

#### 4.3.3. Cell-Type-Specific and Etiological Tests in Experimental Models

Experimental models are required to test the proposed predominance of PV-centered dysfunction. In a well-learned forelimb task, cell-type-specific recording and manipulation could compare the behavioral consequences of reducing PV-, SST-, or VIP-centered inhibitory control within a defined motor cortical ensemble. The framework predicts that selective disruption of rapid PV-centered inhibition will be especially likely to produce excessive, spatially diffuse, and threshold-dependent output while preserving lower-demand performance.

Failure of PV manipulation to produce this phenotype would challenge the proposed cellular mechanism. The claim of PV predominance would also require modification if disruption of SST- or VIP-centered circuitry reproduced the same threshold-dependent and sustained abnormal output as consistently as PV disruption. Conversely, selective restoration of PV recruitment or inhibitory output should increase the task demand required to elicit abnormal motor activity.

A separate experimental design should test the proposed etiological sequence. Animals could first be trained to stable performance on a skilled motor task and then exposed to an altered coordination requirement that reduces effective task capacity. One group would practice only within its retained functional range, whereas another would be repeatedly driven above that range. Longitudinal recordings of pyramidal and identified inhibitory populations could determine whether altered coordination first reduces task capacity and whether subsequent overreaching produces progressive divergence between excitatory and inhibitory circuit efficacy.

The framework predicts that overreaching, rather than altered coordination alone, is required for formation of a threshold-dependent hyperexcitable configuration. Equivalent abnormalities in both groups would argue against overreaching as the decisive factor, while development of abnormal output without a preceding reduction in functional capacity would challenge the proposed sequence from partial baseline shift through true weakness to dystonia.

A subsequent BATR-like phase could test reversibility. Animals would practice within the intact performance range while behavioral thresholds and excitatory and inhibitory physiology are tracked longitudinally. The strongest support for the framework would consist of a linked sequence in which altered coordination reduces capacity, overreaching produces excitatory-dominant activity, a behavioral symptom-threshold emerges, and symptom-free retraining restores inhibitory control while raising that threshold. Partial or divergent results would identify which elements of the etiological and rehabilitation hypotheses require modification.

### 4.4. Translational Implications and Proposed Clinical Evaluation of BATR

BATR is presented as a rehabilitation hypothesis rather than a validated treatment protocol. Its operational principle is that practice should remain within a range in which the intended movement is produced accurately and without overt dystonia or true weakness. Clinical development will therefore require standardized procedures for threshold assessment, training dosage, progression, monitoring, and safety.

The first requirement is a reproducible pre-BATR procedure for estimating the relevant performance boundary. Initial validation studies should prespecify the affected task, posture, apparatus, instructions, primary task-demand variable or fixed multidimensional demand index, and criteria for accurate performance, task-specific output limitation, and overt dystonia. After familiarization, task demand should be advanced from clearly intact levels toward the anticipated boundary under standardized conditions. An adaptive staircase or Bayesian psychometric procedure could concentrate observations near the provisional boundary while reducing trials far from the clinically relevant range [79]. Prespecified stopping criteria should limit the number and duration of trials in which overt dystonia or output divergence is elicited. Dedicated validation studies could compare ascending and descending series to quantify hysteresis and could use randomized presentation to estimate order, anticipation, and fatigue effects. The presence or absence of overt dystonia should be determined using prespecified behavioral criteria, ideally combining blinded video ratings with task-appropriate kinematic and surface-electromyographic measures. A psychometric function relating task intensity to the probability of overt dystonia could then be used to estimate the individualized symptom-threshold and its uncertainty.

The true-weakness threshold should be estimated separately from the relationship between attempted task demand and realized output. Attempted demand should preferably be externally specified, such as through a metronome rate, force target, movement-amplitude target, or acoustic-intensity target, rather than inferred solely from subjective effort. The relationship between target demand and realized output should first be characterized within the intact lower-intensity range. Segmented or change-point analysis could then identify the highest intensity before realized output departs reproducibly from that lower-intensity scaling relationship beyond a prespecified performance tolerance or measurement-error band. Repeated confirmation near the estimated breakpoint would be required to distinguish a task-specific output limitation from an isolated poor trial.

Reliability should initially be evaluated during a no-training validation phase that precedes formal BATR. This phase is intended to establish the measurement properties of the threshold-assessment procedure and the initial conservative practice range; it is not part of routine BATR. Repeated runs within the same session and repeated sessions on separate days should be conducted under otherwise stable conditions. Relative test–retest reliability of the resulting threshold estimates should be quantified using an absolute-agreement intraclass correlation coefficient selected according to the study design and reported with its confidence interval. Absolute measurement error should be characterized using the within-participant standard deviation and, where the measurement scale permits ratio interpretation, the intra-individual coefficient of variation. Studies should also report the standard error of measurement, minimal detectable change at a prespecified confidence level, and Bland–Altman bias and limits of agreement adapted for repeated measurements [80,81,82,83]. Inter-rater and intra-rater agreement for the classification of overt dystonia should be assessed separately. A hierarchical mixed-effects model could additionally partition variability attributable to participant, session, trial, presentation direction, and rater. Medication timing, recent practice, sleep, pain, fatigue, attention, and stress should be standardized where possible and otherwise measured as potential sources of within-person variability. If ascending and descending thresholds differ systematically, they should be reported separately rather than averaged without qualification.

After baseline calibration, formal BATR would proceed without deliberate threshold searching or repeated symptom provocation. Practice would remain at or below a conservative working intensity defined by the symptom-threshold in individuals without a measurable true-weakness range and by the lower of the symptom-threshold and true-weakness threshold where both are present. Day-to-day progression could be guided by a proposed participant-reported progression cue: a spontaneously perceived expansion of the intact functional range during otherwise routine practice. This may take the form of a greater symptom-free movement intensity, a greater attempted intensity with preserved translation into realized output, or both. Greater realized output at a comparable attempted demand may provide an additional manifestation in individuals with true weakness. Such gains may emerge within or between sessions and need not occur continuously.

Participants would not be instructed to test a prespecified higher intensity or locate either threshold. If a greater performance level emerges spontaneously and remains accurate, free of overt dystonia, and functionally intact during subsequent practice, it may become the new working intensity. Overt dystonia, task-specific underproduction, divergence between attempted intensity and realized output, disproportionate effort, or compensatory movement would indicate that the level is not reliably within the intact range and should prompt a return to the last stable working intensity.

The participant-reported progression cue is a testable clinical hypothesis rather than a validated progression criterion. Prospective studies should record its timing and circumstances through structured practice logs and, where feasible, passive task-performance measures such as movement speed, force, pressure, amplitude, acoustic intensity, or kinematics. Formal threshold reassessment should occur after a prespecified BATR block and at follow-up, separate from routine practice. These assessments should determine whether the symptom-threshold, true-weakness threshold, or both shifted beyond the relevant measurement-error or participant-specific uncertainty margin and whether the reported form of improvement predicted the corresponding objective change. Failure of the cue to predict measured change, or frequent reports of improvement without preserved symptom-free performance or faithful output realization, would argue against its reliability as a progression signal. Formal reassessment would therefore evaluate BATR efficacy and the progression rule rather than guide individual practice sessions.

The framework does not yet justify fixed prescriptions for daily practice duration, uninterrupted session length, repetition count, rest intervals, or progression rate. Dose-finding studies should compare session length, distributed versus concentrated practice, rest schedules, and clinician-supervised versus partially self-directed formats. In partially self-directed formats, investigators should test whether participant-reported spontaneous expansion of the intact functional range can guide progression without encouraging deliberate threshold testing. Initial studies should emphasize feasibility and protocol refinement, including the reliability of baseline threshold estimates, adherence to the prescribed range, frequency of inadvertent symptom provocation, correspondence between reported improvement and passively measured performance, and adverse events such as fatigue, pain, compensatory movement, deterioration in other tasks, and treatment burden.

Subsequent controlled trials could compare manualized BATR with usual care, an active motor-training control, or BATR combined with established symptomatic treatment. Outcomes should include blinded clinical ratings, quantitative task kinematics, patient-reported function, pre- and post-training measurements of the symptom-threshold and, where present, the true-weakness threshold, correspondence between participant-reported progression cues and objective performance change, and durability after training is reduced or discontinued. The framework predicts that reducing or discontinuing BATR would primarily slow or halt further improvement rather than immediately reverse previously consolidated gains, although this prediction requires prospective testing. Mechanistic measures such as SICI, surround inhibition, sensory processing, and network-level imaging could determine whether clinical improvement follows the physiological route predicted by the framework.

Formal clinical integration should be considered only after controlled studies establish efficacy beyond natural fluctuation, expectancy effects, nonspecific practice, and compensatory task modification, together with acceptable safety, dosage, and durability.

## 5. Conclusions

FTSD is hypothesized here to arise within an M1 TSMS in which excitation becomes disproportionately strong relative to rapid PV-centered inhibitory control. In the proposed etiological sequence, altered movement coordination reduces the capacity of a previously consolidated functional synergy, creating a task-specific true-weakness range. Repeated overreaching beyond that capacity then biases plasticity toward excitation, driving the formation and consolidation of a dystonic synergy and producing a symptom-threshold at which the dystonic configuration becomes preferentially recruited.

BATR follows directly from this proposed mechanism. Practice confined to the intact, symptom-free range is predicted to preserve coherent functional-synergy recruitment, strengthen rapid inhibitory control, and progressively raise the symptom-threshold. The reduced spiking model supports the conditional plausibility of an input-dependent crossover between specified functional and E/I-imbalanced conditions, but it does not establish the biological origin of the imbalance, M1 or PV specificity, or BATR efficacy.

The TSMS framework is intended as a falsifiable causal hypothesis. Its central claims can be tested through longitudinal measurement of task thresholds and inhibitory physiology, cell-type-specific manipulation of motor cortical circuits, experimental comparison of within-capacity practice and overreaching, temporal analysis of M1 and extra-M1 changes, and controlled evaluation of BATR. These tests should determine which components of the proposed sequence are supported, which require revision, and whether the framework can distinguish initiating abnormalities from downstream adaptations in FTSD.

## Figures and Tables

**Figure 1:**
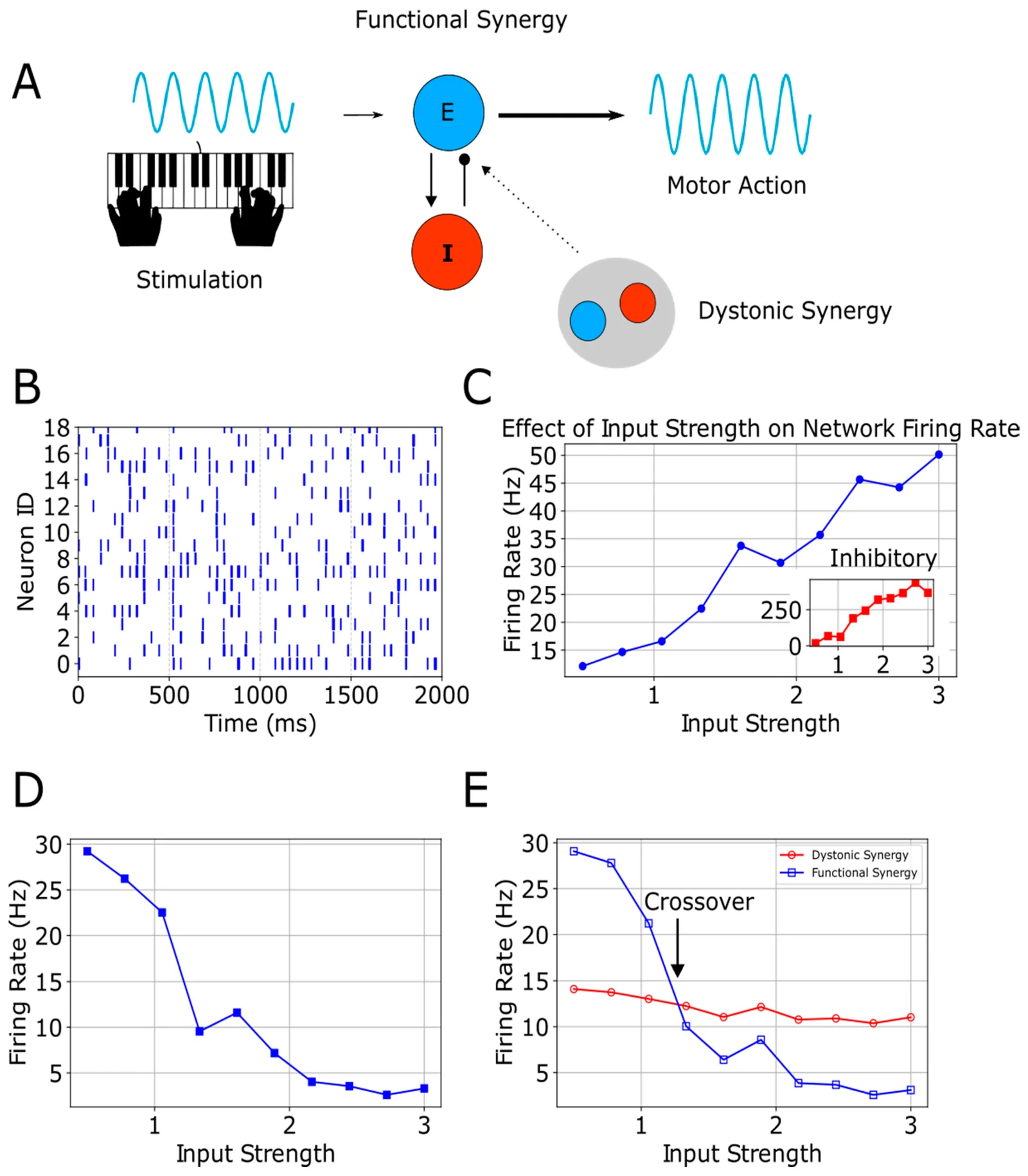
(**A**) **Conceptual schematic of the model framework.** The piano keyboard represents abstract task-related stimulation, and the functional and dystonic synergy labels denote model constructs rather than directly measured biological circuits. The depicted “motor action” is conceptual and is not explicitly simulated. (**B**) Representative raster plot from the functional reference condition. Each row represents a model neuron, and each tick mark indicates a spike. (**C**) Relationship between input strength and network firing rate in the functional reference condition. The inset shows activity of the generic inhibitory model units. (**D**) Alternative model response in which firing rate decreases as input strength increases under a different imposed E/I configuration. (**E**) Comparison of population-averaged firing rates of the FS and DS populations across increasing input strength. The marked crossover denotes the model-level point at which the relative firing-rate ordering changes. Input strength is an abstract model quantity and is not directly calibrated to clinical movement intensity.

**Figure 2:**
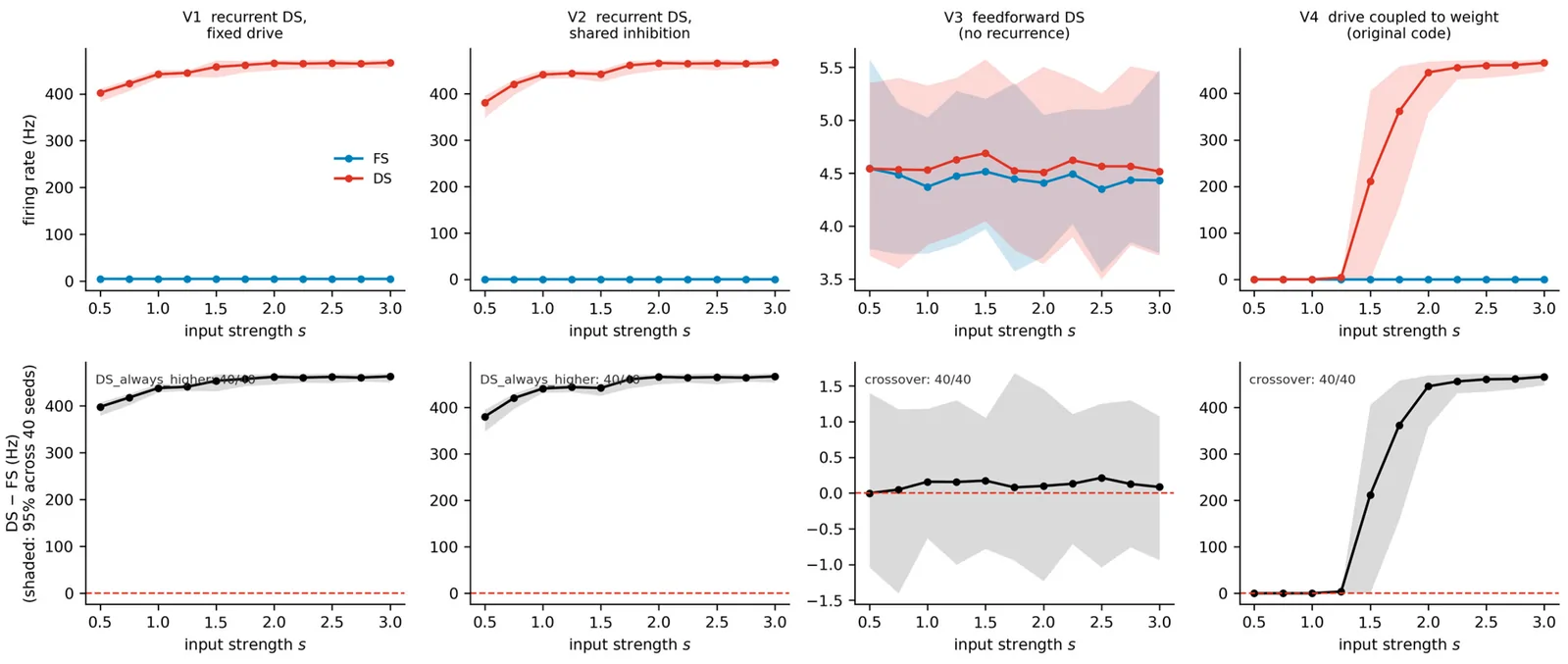
**Replication of the population comparison across random seeds and structural variants.** Top row: population-averaged firing rates of the FS (blue) and DS (red) populations as a function of input strength s, for four structural variants of the model. Lines show the mean across 40 independent random seeds; shaded bands show the 2.5th to 97.5th percentile across seeds. V1, DS population with recurrent excitation and no inhibition, external drive amplitude specified independently of synaptic weight; V2, as V1 with the DS population additionally receiving inhibition from the FS interneuron pool; V3, DS population receiving external drive only, without recurrent connections; V4, as V1 but with each population’s external drive amplitude set equal to its own excitatory synaptic weight, reproducing the behavior of the originally reported implementation. Bottom row: the difference DS − FS for the same simulations, with the zero line dashed in red; annotation gives the number of seeds in which a reversal of ordering was detected within the tested range. Note the differing vertical scales; the refractory ceiling implied by τ_ef = 2.0 ms is 500 Hz.

**Figure 3:**
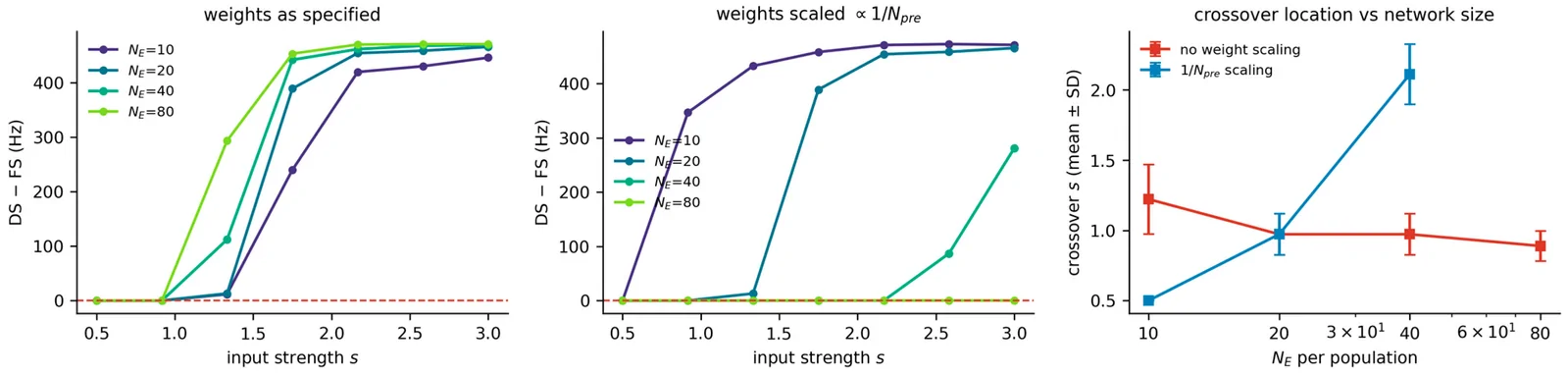
**Network-size dependence of the transition point.** (**Left**): Difference DS − FS as a function of input strength for excitatory population sizes N_E = 10, 20, 40 and 80 (interneuron count N_E/4), with synaptic weights held at their Table 2 values; means across 15 seeds. (**Middle**): The same simulations with synaptic weights scaled in proportion to 1/N_pre so that total synaptic drive per cell is invariant to network size. (**Right**): Input strength at which the ordering reverses, as a function of network size, for both weight conventions (mean ± SD across 15 seeds). No reversal occurred at N_E = 80 under the 1/N_pre convention, so no point is plotted. All panels use structural variant V4.

**Figure 4:**
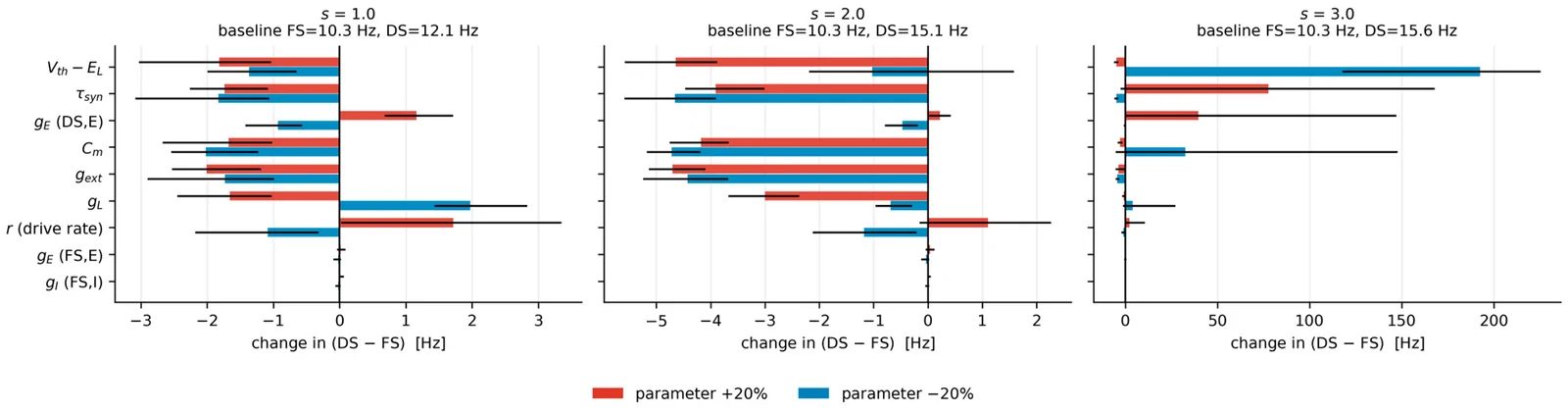
**One-at-a-time sensitivity of the population difference, evaluated at three operating points.** Tornado plots showing the change in the difference DS − FS produced by perturbing each parameter by +20% (red) or −20% (blue) with all other parameters held fixed, in the non-saturated regime identified by grid search. Horizontal black lines give 95% intervals across 30 seeds. Panels correspond to input strengths s = 1.0, 2.0 and 3.0; baseline firing rates of both populations are given above each panel. Sensitivity is expressed as an absolute change in Hz rather than as a percentage, because percentage change is undefined for a quiescent population and uninformative for a saturated one. Parameters are ordered by the magnitude of their effect at s = 3.0. r, external Poisson drive rate; τ_syn, synaptic conductance decay time constant; C_m, membrane capacitance; g_L, leak conductance; V_th − E_L, rest-to-threshold distance; g_ext, external drive amplitude; g_E(FS,E) and g_I(FS,I), excitatory and inhibitory synaptic weights of the FS population, respectively; g_E(DS,E), excitatory synaptic weight of the DS population.

**Figure 5:**
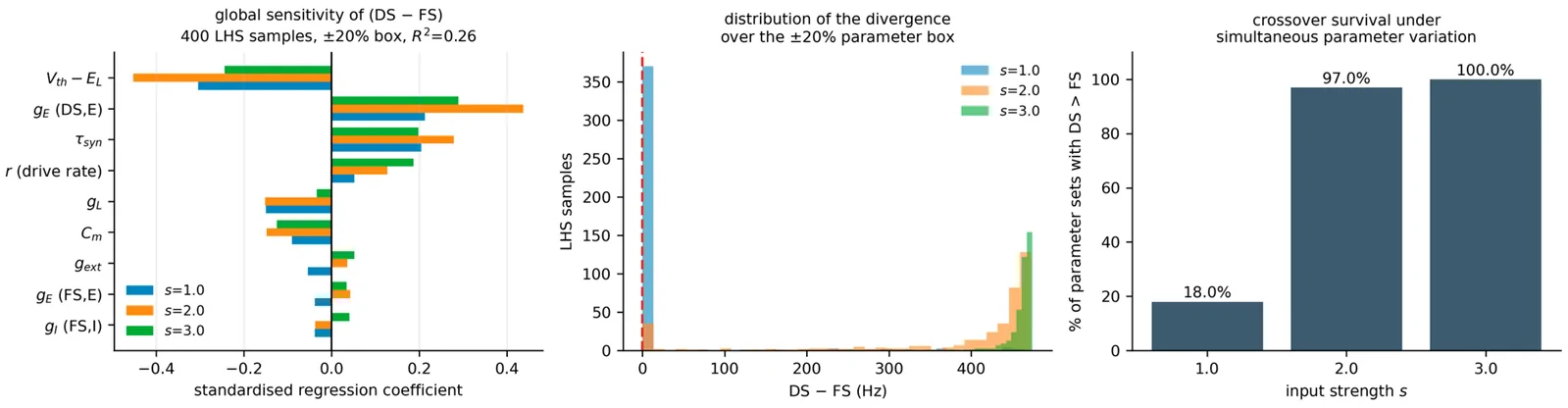
**Global sensitivity analysis with all parameters varied simultaneously.** (**Left**): Standardized regression coefficients for the difference DS − FS, computed from 400 Latin hypercube samples drawn from a ±20% box around the baseline value of all nine parameters, at three input strengths. The coefficient of determination of the linear additive model is given in the panel title. (**Middle**): Distribution of DS − FS across the 400 parameter sets at each input strength; the dashed red line marks equality of the two populations. (**Right**): Percentage of parameter sets in which the DS population exceeded the FS population, by input strength. Structural variant V4.

**Figure 6:**
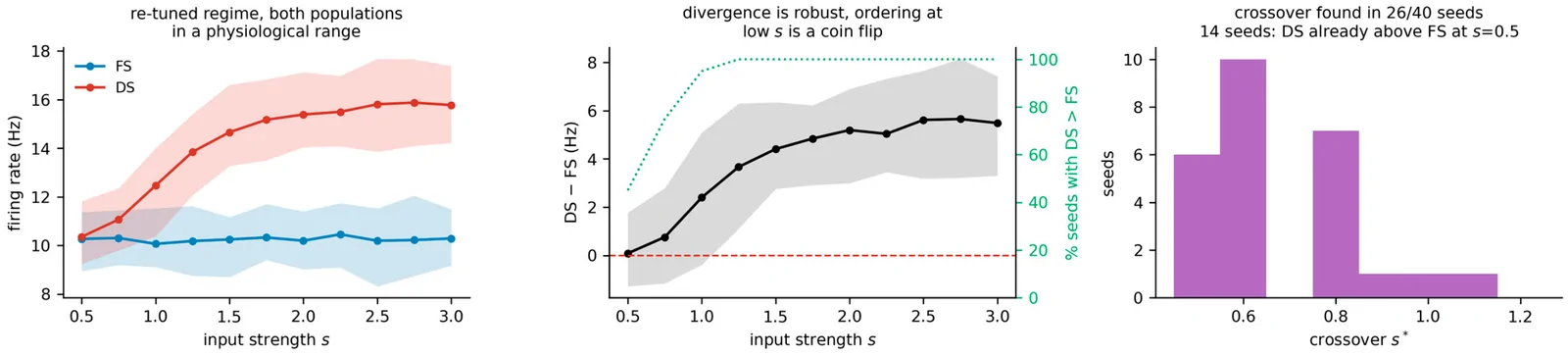
**Behavior in the single non-saturated regime identified by grid search.** (**Left**): Firing rates of the FS (blue) and DS (red) populations as a function of input strength, in the one parameter combination out of 108 tested that placed both populations below 20 Hz while still producing a reversal of ordering; mean across 40 seeds with 2.5th to 97.5th percentile bands. (**Middle**): The difference DS − FS for the same simulations (black, left axis) together with the percentage of seeds in which DS exceeded FS (green dotted, right axis). (**Right**): Distribution across seeds of the input strength at which the ordering reverses; in 14 of 40 seeds the DS population was already above the FS population at the lowest input strength tested and no reversal was detected. s* indicates the crossover positions in previous plots.

**Table 1:** **Key terminology used in the proposed TSMS framework.**

Term	Definition
**Task-specific motor synergy (TSMS)**	A hypothesized task-dependent functional ensemble of pyramidal neurons and local inhibitory interneurons in M1 that contributes to a particular unidirectional movement component within a learned task. The term *synergy* is retained because that movement component may be generated through the coordinated recruitment of multiple muscles or muscle groups. The TSMS is proposed as the cortical organization supporting this coordinated output; it is not itself a muscle-space dimensionality-reduction module or a discrete anatomical unit.
**Functional synergy**	The residual TSMS configuration that supports accurate voluntary performance within the intact range of task demand.
**Dystonic synergy**	A hypothesized excitatory-dominant configuration within the affected TSMS that becomes preferentially recruited above the symptom-threshold.
**Movement intensity**	The task-specific demand placed on the relevant TSMS, expressed through one or more variables such as speed, force, pressure, amplitude, loudness, or intereffector coordination.
**Attempted intensity and realized output**	*Attempted intensity* is the task demand that the individual intends to produce; *realized output* is the movement output that is actually produced.
**Symptom-threshold**	The highest task intensity, under a specified performance condition, at which overt dystonic symptoms remain absent.
**True weakness**	A proposed state of task-specific output limitation in which increasing attempted intensity fails to produce a corresponding increase in realized output despite preserved lower-intensity performance and relative preservation outside the affected task.
**True-weakness threshold**	The highest task intensity at which attempted intensity is still translated faithfully into realized output.
**Overreaching**	Repeated recruitment of a TSMS beyond the level that its current excitatory and inhibitory architecture can implement normally.
**Below- or at-threshold retraining (BATR)**	Systematic practice at or below the lower of the symptom-threshold and, where present, the true-weakness threshold, so that attempted intensity and realized output remain matched and overt dystonia is absent.

**Table 2:** **Single-neuron, synaptic, network, and simulation parameters used in the reported computational analyses.** The source code and associated analysis materials are available at https://github.com/normanklu/Dystonia (accessed on 20 September 2026).

Category	Symbol	Description	Value	Units
**Single neuron (LIF)**	*C*_m_	Membrane capacitance	200	pF
**Single neuron (LIF)**	*g*_L_	Leak conductance	10	nS
**Single neuron (LIF)**	*E*_L_	Resting (leak) potential	−65	mV
**Single neuron (LIF)**	*V*th	Spike threshold	−50	mV
**Single neuron (LIF)**	*V*_reset_	Post-spike reset potential	−60	mV
**Single neuron (LIF)**	*τ*_ref_	Absolute refractory period	2.0	ms
**Synaptic**	*E*_E_ (E)	Excitatory reversal potential	0	mV
**Synaptic**	*E*_I_ (I)	Inhibitory reversal potential	−75	mV
**Synaptic**	*τ*_syn_	Synaptic conductance decay time constant	6	ms
**Synaptic**	*g*_E_—Pop.1 E cells	Fixed excitatory synaptic weight	2	nS
**Synaptic**	*g*_I_—Pop.1 I cells	Inhibitory synaptic weight; scales with input strength s	6 × s	nS
**Synaptic**	*g*_E_—Pop.2 E cells	Excitatory synaptic weight; scales with input strength s	4 × s	nS
**Synaptic**	delay	Axonal conduction delay	1.5	ms
**Network structure**	*N*_ex_	Excitatory neurons per population	20	—
**Network structure**	*N*_inh_	Inhibitory (interneuron) count, Population 1 only	5	—
**Network structure**	*N*_neuron_	Total size of Population 1 (Nex + Ninh)	25	—
**Connection probability**	*p*	The same connection probability is used for all neurons in the network.	1	—
**External Input/Drive**	*r*	Baseline external Poisson input rate, both populations	8	Hz
**Simulation/integration**	*dt*	Integration time step	0.1	ms

## Data Availability

The source code and associated analysis materials supporting the computational modeling and sensitivity analysis presented in this study are publicly available at https://github.com/normanklu/Dystonia (accessed on 20 September 2026). No human-participant or animal data were generated or analyzed in this study.
